# Multipolar, time-dynamical model for the loss compensation and lasing of a spherical plasmonic nanoparticle spaser immersed in an active gain medium

**DOI:** 10.1038/srep33018

**Published:** 2016-09-14

**Authors:** Alessandro Veltri, Arkadi Chipouline, Ashod Aradian

**Affiliations:** 1 Colegio de Ciencias e Ingeniera, Universidad San Francisco de Quito, Quito, Ecuador; 2 Institute for Microwave Engineering and Photonics, Technische Universität Darmstadt, 64283 Darmstadt, Germany; 3 CNRS, Centre de Recherche Paul Pascal UPR 8641, F-33600 Pessac, France; 4 Univ. Bordeaux, Centre de Recherche Paul Pascal UPR 8641, F-33600 Pessac, France

## Abstract

The plasmonic response of a metal nanoparticle in the presence of surrounding gain elements is studied, using a space and time-dependent model, which integrates a quantum formalism to describe the gain and a classical treatment for the metal. Our model fully takes into account the influence of the system geometry (nanosphere) and offers for the first time, the possibility to describe the temporal evolution of the fields and the coupling among the multipolar modes of the particle. We calculate the lasing threshold value for all multipoles of the spaser, and demonstrate that the dipolar one is lowest. The onset of the lasing instability, in the linear regime, is then studied both with and without external field forcing. We also study the behaviour of the system below the lasing threshold, with the external field, demonstrating the existence of an amplification regime where the nanoparticle’s plasmon is strongly enhanced as the threshold is approached. Finally, a qualitative discussion is provided on later, non-linear stages of the dynamics and the approach to the steady-state of the spaser; in particular, it is shown that, for the considered geometry, the spasing is necessarily multi-modal and multipolar modes are always activated.

Metallic nanostructures have since long attracted interest in different fields of nanotechnology, since they can sustain localized plasmon resonances which act as nanoscale concentrators of optical fields. In the presence of optical gain (active medium) near or within the nanostructures, new possibilities open up with the ability to further control, amplify and tune these localized surface plasmon resonances^1^,^2^,^3^,^4^,^5^,^6^,^7^,^8^. The interest of coupling metallic nanostructures with active media culminates in the concept of the “spaser” (Surface Plasmon Amplification by Stimulated Emission of Radiation, the plasmonic equivalent of a laser), which widened their potential applicability to nanoscale lithography, probing, microscopy, optoelectronics and more^9^,^10^. In order to effectively shape optical energy to the nanoscale according to our needs, it is of high importance to dispose of accurate theoretical descriptions taking fully into account not only the effects of the metal to active medium coupling and the associated dynamics, but also, on equal footing, the effects of the nanostructure geometry which governs the spatial structure of the spaser field. In this report, we propose a semi-classical model describing the coupling of the temporal quantum dynamics of the gain elements with a homogeneous, spherical nanoparticle (NP), taking into account not only the lowest, but also all other higher electromagnetic modes of the NP as well.

The past few years have seen a number of works on the optical properties of gain-assisted nanoparticles (NPs) with various geometries: spheres, core-shell, multiple core-shell, ellipsoids…, using more or less refined analytical descriptions, or numerical simulation tools like the finite-element method^3^,^11^,^12^,^13^,^14^,^15^,^16^. All these works were carried out in stationary regimes for the NP and the gain (assuming flat, or Lorentzian, emission lineshapes for the latter). While such a simplification is acceptable far from the lasing threshold and it has been successfully used to describe experimental results^17^,^18^,^19^, it has to fail near and above it, making the obtained predictions inconsistent: gain saturation or complex time-dependent effects may rise, begging for a more elaborate dynamical and non-linear description of the system.

Time-dependent approaches were also developed but are more scarce, starting with the seminal work of Bergman and Stockman introducing the spaser^20^, and then in subsequent works^4^,^21^,^22^,^23^,^24^. Nevertheless, often these approaches are incomplete: either space-dependent modes were not considered for the sake of generality, or they were averaged out through homogeneization, or only the dipolar mode was considered, leaving out all the effects related to the specific geometry of the actual nanoresonator used to generate the spasing. A few purely numerical works have integrated all time and space-dependent effects using two or four-level population dynamics for gain carriers (see ref. 2 and references therein) locally coupled to Maxwell equations, but they were only concerned with so-called “fishnet” geometries, unrelated to NP-based spasers. Moreover, powerful as they are, full-wave simulations do not necessarily allow for a deep understanding of the mechanisms at work, and a complementary model-based approach is undoubtedly useful. Finally, ref. 25 provides an analytical, time-dependent study of a NP in a gain core-metal shell configuration, where only the dipolar mode can exist.

In this article, we consider a single, homogeneous, metallic nanosphere (NP) of radius *a* (as illustrated in Fig. 1), immersed in a gain medium consisting of a dielectric host in which active elements are dispersed (e.g., a laser dye or quantum dot solution). This is the simplest imaginable geometry for spasing, but we will show that it gives rise to rich effects nonetheless. We emphasise that the aim of this work is not to study and optimize a given design for experimental spaser realizations, but rather to lay some understanding of basic physical effects that may arise in spaser systems across various geometries. Still, the situation of homogeneous plasmonic spheres immersed in a gain medium is of experimental relevance to some existing experimental studies^26^,^27^.

The model we present is combining together aspects treated separately in the literature, namely: (*i*) taking into account the appropriate electromagnetic response of the NP, as induced by its specific geometry and inclusive of all multipolar fields; (*ii*) including the population dynamics of quantum emitters, in order to explore the time-dependent behavior of the NP-cum-emitters system; (*iii*) integrating interaction with a sea of emitters around the NP, which is coupled to the spatially inhomogeneous local field created by the NP.

The system will be studied in conditions where it is either excited by a probe field linearly polarized along the *z*-axis (with unit vector 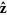): 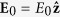, or it is left isolated (**E**_0_ = **0**). Note that **E**_0_ refers to the value of the exciting field taken *inside* the active medium. We refer to the region inside the spherical inclusion as region 1 and to the region outside as region 2, with respective electric fields **E**_1_ and **E**_2_ and respective polarizations **P**_1_ and **P**_2_. The system is taken in the quasi-static limit: 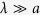 where *λ* denotes the exciting probe wavelength, so that **E**_0_ is assumed to be uniform over the NP.

## Metallic Nanoparticle and Active Medium Description

We start by describing how the field **E**_1_(**r**, *t*), where **r** is the spatial coordinate with origin at the particle center and *t* is time, acts on the electrons in the metallic nanoparticle. We describe this interaction using a free-electron model^28^:


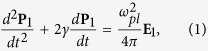


where the polarization **P**_1_ = *n*_*e*_*e***d** is produced by the displacement **d** of the electron cloud with respect to the equilibrium position (*n*_*e*_ is the electron density, *e* is the electron charge); *γ* is the ionic collisions friction coefficient. We also used the plasma frequency 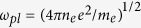 with *m*_*e*_ the electron mass. Assuming *e*^−*iωt*^ harmonic notation for fields, Eq. 1 classically gives back the Drude formula for the metal’s relative permittivity:


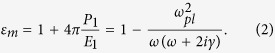


In the following, we will focus on the metal, silver, using experimental data from ref. 29 for numerical values of *ε*_*m*_. Values for *ω*_*pl*_ and *γ* are found by fitting Eq. 2 against these data (also adding a constant offset *ε*_∞_ to account for non-Drude contributions seen in the experimental measurements). We find: *ħω*_*pl*_ = 9.6 eV and *ħγ* = 0.0228 eV.

We now consider how the field **E**_2_ interacts with the active medium outside the nanoparticle, which is described as a continuum of two-level emitters in a thermal bath using the the optical Bloch equations and the density matrix formalism^4^,^30^,^31^:


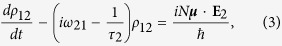



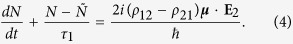


Here *ρ*_*ij*_ is the *i*, *j* element of the density matrix; 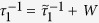 is an effective relaxation rate, with *W* the phenomenological pumping rate, and 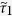 and *τ*_2_ the time constants associated with energy (spontaneous emission) and phase relaxation processes due to the interaction with the thermostat; *ω*_21_ is the transition frequency between levels 1 and 2; *N* = *ρ*_22_ − *ρ*_11_ is the population inversion, where *ρ*_22_ and *ρ*_11_ are the diagonal matrix density elements, and finally 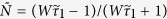. The term ***μ*** · **E**_2_ accounts for the non-radiative coupling to the metal, with ***μ*** the transition dipole moment, while the radiative contribution is taken into account in Eq. 4 through the *τ*_1_ relaxation time. Importantly, note that all quantities in Eqs 3 and 4 are taken implicitly as both space and time-dependent (functions of **r** and *t*).

With *χ*_*b*_ the susceptibility of the background (passive) dielectric host, and *n* the number density for the active elements (e.g., dye molecules), the space and time-dependent polarization of region 2 is obtained by averaging over the dipole moments of the gain elements, assuming they are randomly oriented with respect to the field **E**_2_ (due to Brownian motion in the host solution):


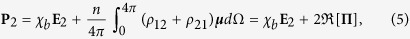


where Ω denotes solid angle.

We have defined the new quantity **Π** as


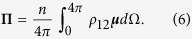


Physically, the role of **Π** is to keep track of the part of the polarization in medium 2 contributed to by the emitters. Integrating the system of equations 3 and 4 over solid angles, we obtain:


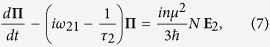



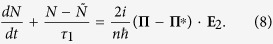


We next use the rotating waves approximation, assuming that the frequency of the probe field *E*_0_ is near-resonant 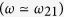 and assuming an *e*^−*iωt*^ harmonic form for all time-dependent quantities. The system to solve becomes:


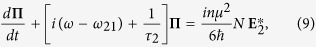



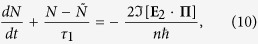



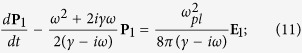


together with


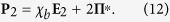


(Now **Π**, **E**_1,2_ and **P**_1,2_ from here on denote only the slowly-varying enveloppes of previously introduced physical quantities.) It is worth noting that the population inversion *N* = *N*(*r*, *θ*, *t*) as it appears in this system of equations is, in the general case, non-uniform due to the non-uniformity of the source term appearing in the r.h.s. of Eq. (10).

We can now in particular calculate the permittivity *ε*_*h*_ of the active medium under pumping (without particle), when excited by a uniform field **E**_2_ = **E**_0_: using Eq. (12), and the steady state-value of **Π** extracted from Eq. (9), one finds a Lorentzian lineshape centered on *ω* = *ω*_21_,


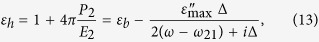


where *ε*_*b*_ = 1 + 4*πχ*_*b*_ is the permittivity of the background medium, 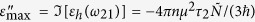 is the maximum level of gain and Δ = 2/*τ*_2_ is the emission linewidth. Such Lorentzian emission lineshapes have been widely used in classical steady-state models for plasmon-gain interaction^3^,^11^.

A metallic nanoparticle such as the one studied here naturally has a dipolar localized plasmon resonance frequency *ω*_0_, which can be obtained from the permittivities *ε*_*m*_ and *ε*_*h*_ just calculated through the standard Fröhlich condition^11^,^32^:


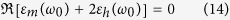


We will from here assume that the gain lineshape is centered exactly at the plasmon frequency, i.e., that *ω*_21_ = *ω*_0_. Note that, while this ensures a better efficiency of coupling between gain and metal, it is by no means a requirement for the effects described below to occur.

## Multipolar Mode Equations and Boundary Conditions

We now look into describing the complete system composed of the NP immersed in the active medium. We start by assuming that the probe field amplitude *E*_0_, as well as *E*_2_, are sufficiently small that the right-hand-side term 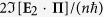 of equation 10, which measures the rate at which the population inversion of the gain elements is depleted, will remain negligible. This means that the population inversion, according to Eq. (10), remains spatially uniform: *N*(*r*, *θ*, *t*) = *N*_0_(*t*). (Situations where this approximation does not hold anymore will be discussed in the last Section of this Report.)

Due to the spherical geometry of the problem, we introduce spherical harmonics, i.e., multipolar polarization modes of the particle and electromagnetic fields. Introducing the potentials *ϕ*_1,2_ and *ψ*_1,2_ such that 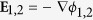, 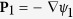 and 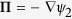, one can look for solutions of the Laplace equations for potentials in terms of a superposition of multipolar modes which are obtained by expanding the angular dependency on the Legendre polynomials 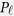. Using spherical coordinates centered on the NP, with *r* = |*r*| as the radial distance, and *θ* as the polar angle, taking into account that the potentials should be regular at *r* = 0 and that for 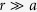, the field has to reconnect to the external, uniform field **E**_0_, the following expressions for *ϕ*_1,2_ and *ψ*_1,2_ are obtained:


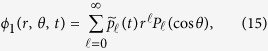



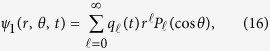



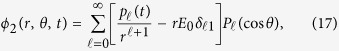



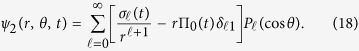


Here, *δ* stands for the Kronecker symbol; 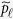, 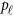 are the mode amplitudes for the electrical fields **E**_1,2_; 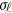 and 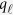 are the mode amplitudes for the polarizations **P**_1_ and **Π**. The quantity Π_0_ is defined as the uniform component of **Π**, reflecting the polarization response (in the *z*-direction) arising from only the active elements in the absence of nanoparticle, when submitted to the probe field **E**_0_.

We can now insert these expansions in system (9–11), to obtain the full set of time-dynamical evolution equations for Π_0_, *N*_0_, 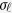 and 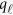 as functions of the probe field *E*_0_:


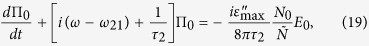



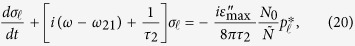



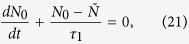



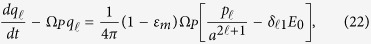


where 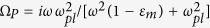, and the dielectric permittivity *ε*_*m*_ of the metal from Eq. 2 were used.

Finally, it is necessary to take boundary conditions at the NP surface (*r* = *a*) into account. The continuity condition for the tangential electrical field writes as 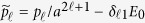, and has been already implemented to obtain Eq. (22). Continuity of the normal electrical displacement links 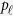 to the variables Π_0_, 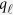 and 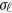, closing the system in the following way:


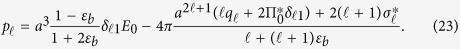


The set of Eqs (19, 20, 21, 22, 23) represents the first complete treatment of a spherical NP immersed in an active gain medium, taking into account the full array of multipolar modes. We are now in a position to consider spasing and amplification effects in the optical response of this system.

## Spasing Threshold and Onset of Instability Under Zero External Field

In a similar way to classical lasing, spasing occurs as a self-oscillation of the electromagnetic field inside and outside the NP, in the absence of an external driving (probe field *E*_0_ = 0), when the gain level in the system reaches a given threshold. The self-oscillation spasing condition is therefore defined as an unstable point.

This will be reflected by the appearance a non-zero outside field *E*_2_, or in other words, by the appearance of (at least) one non-zero multipolar component 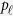. We therefore look for the condition for any given 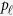 to be non-zero, independently of other modes (*p*_*k*_ = 0, 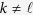). For this, we assume steady-state in Eqs (19, 20, 21, 22) by canceling out all time derivatives, as well as zero external field (*E*_0_ = 0), which directly leads to Π_0_ = 0 and 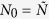. Substituting steady-states values for Π_0_, 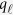 and 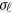 into the boundary condition 23 brings the following equality:


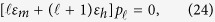


which means that 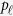 can be non-zero only if


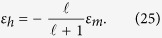


This is the spasing condition for the 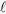-th mode. The imaginary part of the spasing condition yields the value for the gain threshold for the 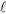-th mode to start self-oscillating: it can be concluded that higher modes require more gain to be triggered. Therefore, the dipolar mode 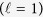 is the one that will be most easily activated:


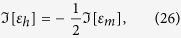


and it is the dipolar threshold that sets the absolute spasing threshold for the NP.

Note that this is the same condition as discussed in ref. 11 for the singularity of the classical dipolar polarizability formula for a sphere (see also further down). We also have analytically checked that this spasing condition corresponds to the point where losses in the system are exactly balanced by the gain, as holds for the threshold of classical lasers.

We now continue with describing the first stages of the spasing instability, right at the onset of oscillations, when the gain value in the active medium exceeds the above threshold. Because the dipolar mode has the lowest threshold, it will initially have the fastest growing rate among all unstable modes for a given gain value, and hence is expected to dominate this onset.

We therefore consider a dipolar approximation to the set of equations (19, 20, 21, 22, 23), valid as long as all modes with 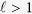 are negligible. In this situation, and still assuming *E*_0_ = 0, the system (19–23) reduces significantly:


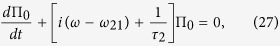



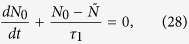



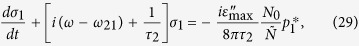



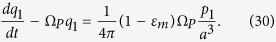


Equations (27) and (28) have trivial solutions, and assuming physically meaningful initial conditions, where Π_0_|_*t*=0_ = 0 (due to the absence of externally imposed field) and 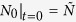 (stable pumping), these variables do not evolve in time: Π_0_(*t*) = 0, 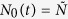. One then only needs to solve the two remaining equations (29) and (30). Using the boundary condition (23) to express *p*_1_, (29) and (30) form a linear system that can be solved analytically:


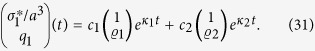


It appears that the temporal dynamics of the system is dictated by two eigenvalues 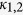:


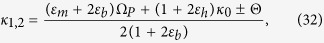


where


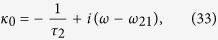


and


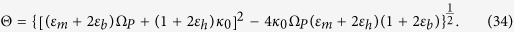


The parameters 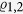 are


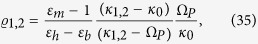


and the constants *c*_1,2_ are related to initial conditions.

**Figure 2:**
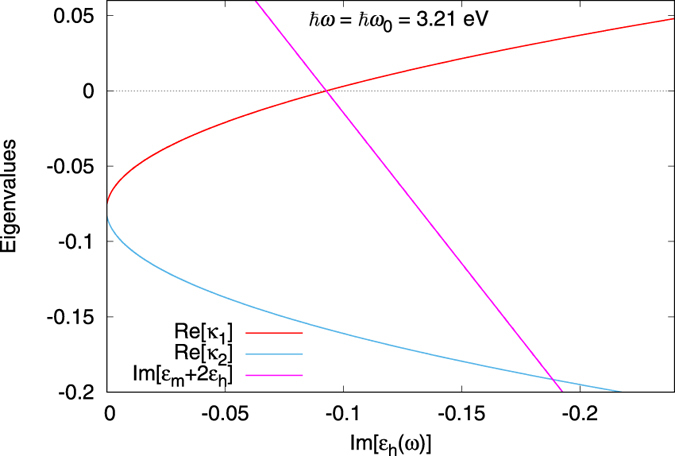
Spasing threshold and eigenvalues 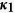 and 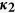: 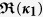 becomes positive (instability) exactly when 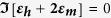 (dipolar spasing threshold). Parameters: *ε*_b_ = 1.85 (ethanol solvent), *ħω*_21_ = *ħω*_0_ = 3.21 eV, *ħ*Δ = 0.2 eV.

The spasing instability (self-oscillation) will arise if at least one of these eigenvalue has a positive real part. Figure 2 shows that indeed the real part of 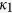 becomes positive exactly when the gain exceeds the spasing threshold, i.e., when 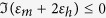 (calculated at the plasmon frequency, *ω* = *ω*_0_). The real part of the other eigenvalue, 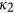, remains always negative.

**Figure 3:**
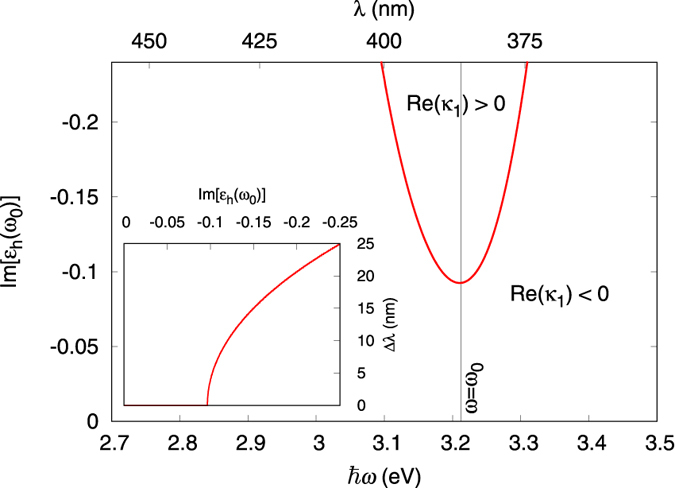
Main figure: map of the stable and linearly unstable region (inside contour), as a function of frequency/wavelength and the overall gain level in the active medium 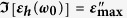. Inset: instability region width Δ*λ* as a function of overall gain. Parameters: *ε*_b_ = 1.85 (ethanol solvent), *ħω*_21_ = *ħω*_0_ = 3.21 eV, *ħ*Δ = 0.2 eV.

It is interesting also to consider the span of the unstable region with 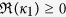 when the overall gain value 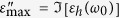 varies: this is mapped in Fig. 3 for a 10 nm-size silver nanoparticle. It is at first quite narrow around the plasmon wavelength 2*πc*/*ω*_0_ = 386 nm (with *c* the celerity of light) when the gain is close to the threshold, but then reaches spectral widths of a few tens of nanometers, as gain is further increased. This provides a rather large frequency range operation for the nanolaser; note, however, that this spectral range should not be confused with the actual linewidth emission of the lasing system: the latter should be calculated in the non-linear steady-state lasing regime, as the full width at half maximum of the emission intensity, and is expected to be much narrower.

Naturally, as the instability develops, the dipolar approximation used in this section will fail: consequences of this failure and later stages are discussed in Section 5 (“Above spasing threshold”) further down. We before consider a situation of practical importance, namely the behaviour of the system when excited by an external field (pump field), both below the spasing threshold and above it, again in the initial stages of the instability.

## Response To An External Field - Linear Amplification Regime

### Below spasing threshold

We now consider the case where a probe field 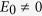 is shone onto the nanoparticle. We begin by considering situations where the overall gain value 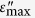 remains below the spasing threshold. Since no field is expected to become extremely strong, we consider as before that 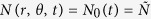.

This brings about two physically important consequences: (*i*) the system (19–22) is linear; (*ii*) only dipolar terms survive 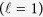 and all multipolar terms 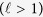 are extinct. The latter property can be inferred upon close inspection of the differential system: modes are coupled within pairs 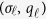 of the same order 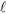 only [Eqs (20, 21, 22)]. Among such pairs, however, only the equations for *σ*_1_ and *q*_1_ exhibit non-zero source terms (proportional to *E*_0_ and Π_0_), stemming from the expression of *p*_1_. Hence, after a very short time, all multipolar terms must naturally decay to zero, except dipolar ones which are fed through the above-mentioned source terms. Finally, it is only necessary to solve the equations for Π_0_, and the two active modes *σ*_1_ and *q*_1_:


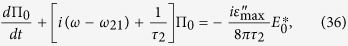



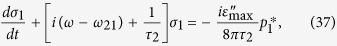



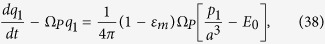


alongside the expression for *p*_1_:


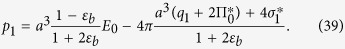


We first look for the (linear) steady states of the system under field; this is best represented by computing the steady-state dipolar polarizability, 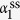, of the sphere, which we classically define as the ratio of the dipolar moment *p*_1_ of the NP to the exciting field^28^: 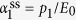. To this purpose, time derivatives are canceled out in the above system, and with the help of (39), we find:


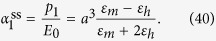


Unsurprisingly, this no other than the well-known quasi-static formula for the dipolar polarizability of a spherical inclusion^28^. This simple formula, where the active nature of the host medium is described by letting *ε*_*h*_ have a negative imaginary part [as per Eq. (13)], was indeed studied by some of us in a previous work^11^ as the simplest step towards loss compensation phenomena: this empirical approach is here revealed as the steady-state, linear approximation of our full time-dynamical model.

**Figure 4:**
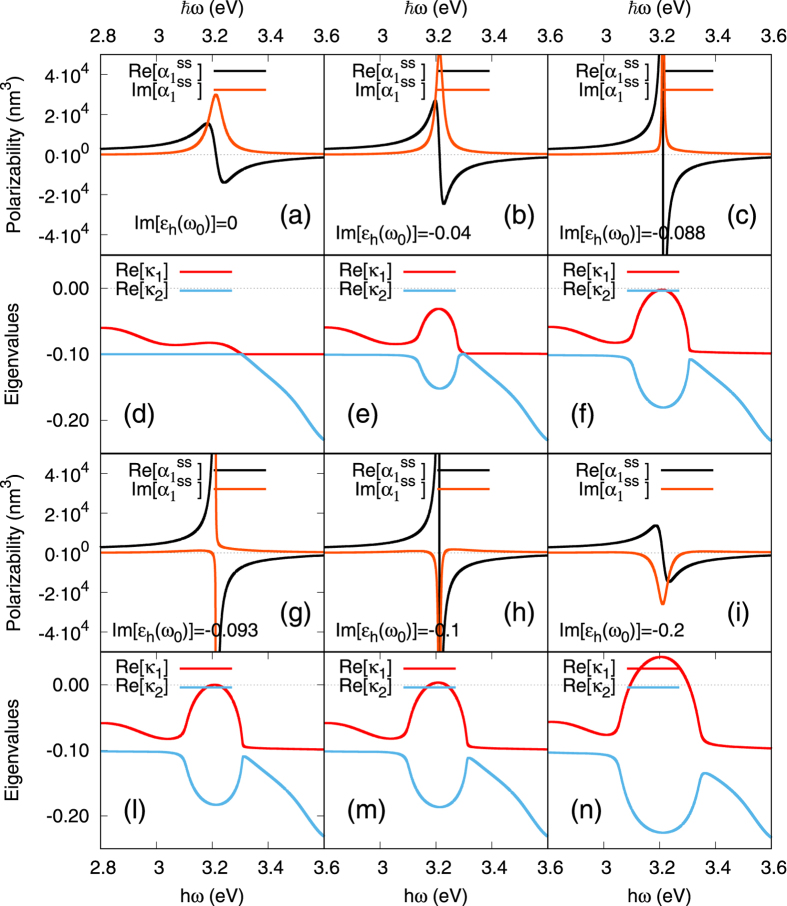
Evolution of the steady-state plasmonic response of a 10-nm silver nanoparticle as gain is increased in the surrounding medium. Parameters: *ε*_b_ = 1.85 (ethanol solvent), *ω*_21_ = *ω*_0_ = 3.21 eV, *ħ*Δ = 0.2. (**a**–**c**,**g**–**i**) Steady-state dipolar polarizability 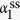; (**d**–**f**,**l**–**n**) Real part of eigenvalues 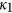 and 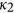, corresponding to each of the polarizability curves.

The evolution of the steady-state 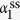 is shown in Fig. 4 for the same 10 nm-silver nanoparticle (see also ref. 11), where the gain level 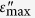 is progressively increased, i.e., goes from zero towards more and more negative values. (Note that we assumed a fixed, large power for the pump: 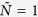; The same behaviors can be obtained by fixing the value for 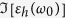 and varying 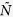 from 0 to 1). We observe that first, the plasmon (here observed as a resonance in the polarizability) is gradually amplified with increasing finesse (Fig. 4a–c): this is what we call the “linear amplification regime” where partial loss compensation occurs. This regime by itself, is a very interesting one in practical applications: it requires much less gain to be introduced, is more easily attained than the spasing one, and to a certain extent, is able to compensate the intrinsic losses found in natural plasmonic resonances.

Then, when the gain value reaches the spasing condition [canceling the denominator in 40], 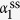 is at its sharpest and formally becomes singular (Fig. 4g). This signals the failure of the used linear, dipolar approximation, and new effects, including saturation, will set in as discussed further down. In particular, the singularity shall disappear and all quantities shall remain finite.

### Above spasing threshold

We now assume that the gain in the host medium is set above the spasing threshold, and that the system is still excited by an external probe field *E*_0_.

Although it does not represent a fully valid solution anymore, it is worthwhile commenting on the behaviour of the steady-state polarizability above threshold: 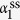 is finite again, but now exhibiting regions in the spectrum where the imaginary part is negative (Fig. 4g–i), a situation which cannot be observed for purely passive systems: this should here be considered as an indication of the appearance of the spasing regime.

The onset of the spasing regime under field can indeed be studied following the same analysis as in zero-field to demonstrate the onset of an oscillation (instability), by solving the linear system (36–38) analytically. One obtains:


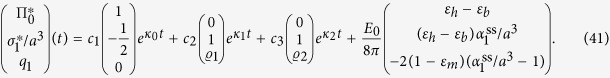


where all parameters are as defined earlier [Eqs (32–35)].

Here again, under field, the spasing instability sets in when 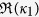 becomes positive (the other eigenvalues 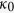 and 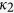 remain always stabilizing), as shown in Fig. 4: for low gain values [(d) to (f)], 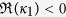; then at the spasing threshold ((g)], 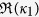 reaches 0 exactly at the plasmon frequency; finally, from (l) to (n), there is an unstable frequency range around the plasmon frequency, where 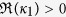; as before, the width of this region shall correspond to the spaser emission width.

**Figure 5:**
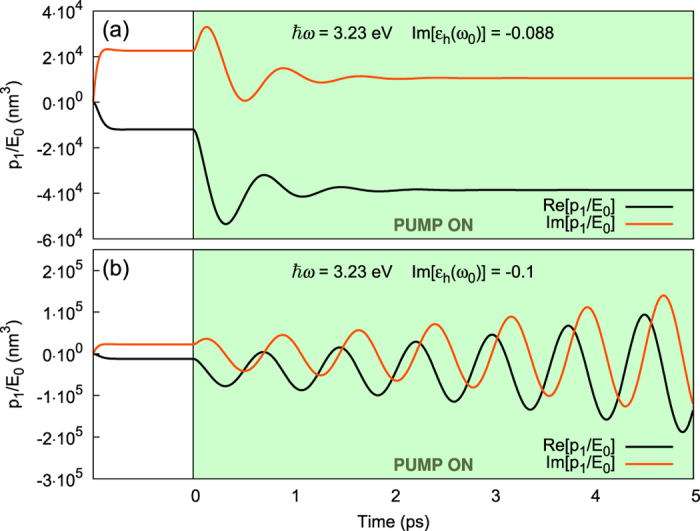
Time evolution of the dipolar moment of the nanoparticle *p*_1_(*t*) of a 10-nm silver nanoparticle. The nanoparticle is under constant excitation from the probe field **E**_0_, while the pump is switched on from *t* = 0 on. (**a**) Example of stable regime (amplifying regime, under spasing threshold). (**b**) Example of onset of the spasing regime (above threshold).

A close inspection of Fig. 4(m,n) (zoomed-in detail not provided) reveals an interesting fact: sign changes in 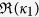 always happen extremely near to sign changes of 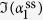, to a precision better than 0.01 eV. Hence, regions of spasing instability very closely coincide with regions with 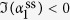 (“active” polarizability), and therefore, in the present system, an easy rule-of-thumb to predict the extent of the spasing regime is to look for sign changes in the imaginary part of the simple formula (40). Obviously, whenever the instability sets in, it prevents the linear steady-state solution represented by 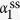 to settle, and the dynamics of the system will become increasingly non-linear as discussed further down. To illustrate the instability, in Fig. 5, we compare the evolution of the dipolar moment *p*_1_(*t*), as calculated from the exact solution 41 for 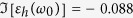 (as in Fig. 4c) and 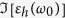 (as in Fig. 4h), both observed at the same point of the energy spectrum (*ħω* = 3.23 eV). For the former, 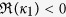 (i.e., below spasing threshold) and therefore a steady-state (in the “amplifying regime”). For the latter, 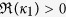 so that the spasing instability sets in with a linear growth rate 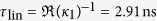, preventing the linear steady state to appear, and preparing for the coming of a full non-linear temporal dynamics as discussed in the next section.

## Non-Linear Regime and The Rise of Higher-Order Modes

We now describe qualitatively the later stages of the spasing instability explored in the previous sections.

At the onset of the spasing instability, the amplitude of the plasmonic dipolar mode grows with a typical rise time 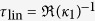, both with and without excitation by the field *E*_0_. For times 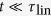, this regime is linear as described in the previous sections.

For times 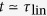, since the dipolar mode has been growing exponentially (and so does *E*_2_), the term 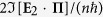 will no more remain negligible in Eq. 10: the instability enters a regime of non-linear growth. Since both **E**_2_ and **Π** are spatially non-uniform fields, Eq. 10 indicates that the population inversion *N* will also take on a non-uniform distribution. In order to account for this, the following expansion for *N* can be introduced:


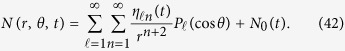


where 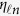 are the mode amplitudes of the population inversion and *N*_0_ is the already presented, spatially uniform (flat) mode. Taking into account this expression transforms the original system (9–11) into a set of non-linear equations displaying intricate cross-coupling terms not present in Eqs (19–22).

The numerical solutions of this system will be explicitly discussed in a forthcoming paper devoted to studying the non-linear amplification regime. Qualitatively, the effect of these new non-linear terms is to couple together 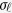 and 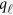-terms of different 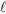-orders via the appearance of the 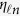 modes. As a consequence, electromagnetic energy will always cascade from the initially unique 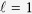 dipolar mode (which benefit from source terms), towards higher (sourceless) modes 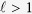, thereby exciting multipoles in the particle.

Therefore we can conclude that, briefly after the spasing instability launches (a few nanoseconds in the example of Fig. 5), the response of the gain-assisted NP always becomes multipolar. Interestingly enough, this is true however small the NP is compared to the probe wavelength, due to local feedback effects with the gain medium. This result means that the response of the spaser, in the geometry studied in this article, is intrinsically a multi-modal one; other geometries for the NP can tailor the response to be exclusively dipolar, for example the “nanoshell” geometry^25^.

Finally, for times 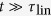, the population inversion will be sufficiently depleted that it will limit the growth of the instability, and a final state will be reached. The exact nature of this long-term state, as it is well-known from the physics of laser oscillations, can be diverse^24^,^33^,^34^: steady-state, multistability, stochastic behaviour, burst-like dynamics, etc. Moreover, in the case where the system is subjected to a non-zero external external field *E*_0_, synchronicity issues will rise between the natural resonant frequencies of the NP oscillator (here, the resonance frequencies of the multipoles) and the forcing frequency^25^,^35^. The exact nature of this final state requires a thorough study, depending on the various model parameters, which is out of the scope of this paper.

In case the transient evolution results in a final stationary state, however, the steady-state values of *N*, Π and *P*_2_ are found easily by canceling time-derivatives in, and solving simultaneously, Eqs (9), (10) and (12). This reveals that the saturated gain medium can be described through a non-linear permittivity^16^,^25^ 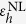, as a function of the local field *E*_2_:


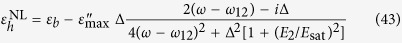


with the saturation field 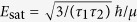.

The non-linear permittivity can then either be used inside numerical simulations^16^ to find the field distribution and other properties of the stationary lasing state, or equivalently, the associated non-linear, steady-state value of *N* can be introduced into our analytical equations (9, 10, 11) to obtain a multipolar mode description of the lasing state.

In conclusion, a full multipolar and dynamical model has been established to describe the optical response of a single metallic nanosphere immersed in an active medium. We have shown that for low gain values in the active medium, the response of the nanoparticle is a steady state and corresponds exactly to the results predicted via the quasi-static classical formulae for dipolar polarizability. This is a regime of amplification, where losses are partially compensated and the plasmonic response is amplified. Then, when gain values exceed a threshold, a spasing instability occurs, where physical quantities such as the dipolar moment of the NP, initially grow exponentially with time. This instability has been demonstrated both in presence, and in absence of an externally-imposed field (i.e., spasing self-oscillation). After a short while, a cascade of non-linear couplings always launches and activates higher-order, multipolar modes, irrespective of how the NP size compares to the exciting (probe) wavelength. Finally, features of the final, non-linear regime were discussed qualitatively.

The importance of geometry for NP-based spasers cannot be overstated, as the nanoparticle’s shape and composition determine the natural “resonator” in the spasing system. In our case of a homogeneous NP in an active medium, our model shows that the optical response of the resonator is unavoidably multi-modal and multipolar, therefore channeling energy into “dark modes” which may or may not suit one needs; then, depending on what type of application is envisioned, various geometries may be sought after, and optimised as a means to control lasing modes of the nano-emitter. Our analysis predicts where in the spectrum and with which specific gain quantity a mode will be activated, allowing in principle to design optical measures aiming at mode-specific amplification regimes, in a very simple geometry.

## Additional Information

**How to cite this article**: Veltri, A. *et al*. Multipolar, time-dynamical model for the loss compensation and lasing of a spherical plasmonic nanoparticle spaser immersed in an active gain medium. *Sci. Rep.* **6**, 33018; doi: 10.1038/srep33018 (2016).

## Figures and Tables

**Figure 1:**
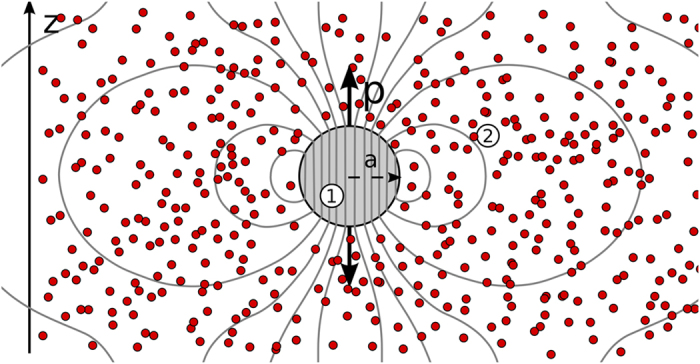
The system under study: a single, homogeneous spherical nanoparticle made of metal (in light gray), immersed in an active host medium made of many active dipolar elements (red dots) dispersed into a background dielectric host.
